# Screening of immune biomarkers in different breeds of chickens infected with J subgroup of avian leukemia virus by proteomic

**DOI:** 10.1080/21505594.2020.1809323

**Published:** 2020-08-30

**Authors:** Fei Ye, Yan Wang, Qijian He, Zhaoshuo Wang, Enyue Ma, Shiliang Zhu, Heling Yu, Huadong Yin, Xiaoling Zhao, Diyan Li, Hengyong Xu, Hua Li, Qing Zhu

**Affiliations:** a Farm Animal Genetic Resources Exploration and Innovation Key Laboratory of Sichuan Province, Sichuan Agricultural University, Sichuan, China; b Guangdong Provincial Key Laboratory of Animal Molecular Design and Precise Breeding, Foshan University, Guangdong, China

**Keywords:** ALV-J, data-independent acquisition, proteomic, chicken

## Abstract

Avian leucosis (AL) is a disease characterized by tumors and is caused by the avian leukosis virus (ALV). Because of the high variability of viruses and complex pathogenic mechanisms, screening and breeding J subgroup of ALV (ALV-J) resistant avian breeds is one of the strategies for prevention and treatment of AL, thus screening of significant immune markers is needed to promote the development of disease-resistant breeds. In this study, data-independent acquisition (DIA) technology was used to detect the DEPs of three breeds of chicken according to different comparison to investigate the potential markers. Results showed special DEPs for spleen development of each breed were detected, such as PCNT, DDB2, and ZNF62. These DEPs were involved in intestinal immune network used in production of IgA signaling pathways and related to immune response which can be used as potential markers for spleen development in different breeds. The DEPs such as RAB44 and TPN involved in viral myocarditis, transcriptional misregulation in cancer, and tuberculosis can be used as potential markers of spleen immune response after ALV-J infection in chickens. Pair-wise analysis was performed for the three breeds after the infection of ALV-J. The proteins such as RFX1, TAF10, and VH1 were differently expressed between three breeds. These DEPs involved in antigen processing and expression, acute myelogenous leukemia, and viral carcinogenesis can be used as potential immune markers after ALV-J infection of different genetic backgrounds. The screening of potential markers at protein level provides a strong theoretical research basis for disease resistance breeding in poultry.

## Introduction

Avian leukosis virus (ALV) is a tumor-causing pathogen that causes great harm to the poultry breeding industry in China [1,2]. The occurrence of Avian leucosis (AL) has become increasingly common in China [3–5]. Therefore, research into ALV via various technologies is vital for poultry development. ALV is a double-stranded RNA virus that not only causes tumors in multiple tissues but also causes immune suppression [4,6]. Studies have shown that the pathogenic rate of J subgroup of ALV is different in chickens of different ages and strains. After experimental inoculation, the incidence of disease varies, which is greatly related to the viral strain, chicken breed, inoculation route, dose and chicken’s age [7,8]. With the development of genomics, transcriptome, proteomics, and bioinformatics, a lot of research into the pathogenic mechanisms of ALV-J, the evolution of the virus, and the resistance to ALV-J in chickens have been done at the macro level [9,10].

The occurrence of certain functions and/or the formation and development of diseases in an organism may be reflected in changes in the abundance of protein content. Data-independent acquisition (DIA) can be used to quantify on a large scale while maintaining targeted protein quantitative technical accuracy and sensitivity advantages [11]. The regulatory mechanism of protein level of ALV-J is little [12–14]. Improvements in the accuracy and depth of proteomic technology have allowed more research into the pathogenic mechanisms and molecular regulatory network of ALV-J. However, due to variabilities in virulence, the omics results of various studies are not suitable for integrated analysis. Therefore, studying omics differences in virus-infected organisms from different genetic backgrounds will provide a strong theoretical basis for breeding disease resistance in poultry. The purpose of this study was to examine the sensitivity of different breeds of chickens infected with ALV-J and use DIA technology to detect differentially expressed proteins (DEPs) in the spleens of these chickens.

## Materials and methods

### Animals for proteomics analysis

All animal care and experimental procedures were reviewed and approved by the Animal Care and Use Committee (#YYS130125) of Animal Care Advisory at Sichuan Agricultural University. This study was carried out in strict accordance with the Regulations for the Administration of Affairs Concerning Experimental Animals of the State Council of the People’s Republic of China. Since the NX0101 strains were from broiler breeds [15], susceptible breeds of Avian broilers were added as controls. Avian broilers (A) were provided by the Zheng Da Company, Chengdu, China. Tibetan chickens (T) were provided by Maoxian farm, Aba, China. Pengxian yellow chickens (P) were provided by Tianhua Company, Chengdu, China. The hens were maintained separately in two pathogen-free negative pressure isolators for poultry (Strong Star Equipment Technology Co, Qingdao, China), and fed according to the chicken feeding management procedures. All chickens used in this study were detected by PCR technology (the specific primer sequences were F: 5ʹ-GCTGCCATCGAGGTTACT-3ʹ; R: 5ʹ-AGTTGTCAGGGAATCGAC-3ʹ) and ALV test kit (IDEXX, Westbrook, USA) to make sure exogenous ALV was free.

### Sample preparation and collection for proteomics analysis

After hatching, the female chicks at 1 day age of three breeds were both divided into injected virus groups and control groups. Each chick of injected virus group was injected with 100 μl ALV-J based on the TCID_50_ of the virus (approximately 10^4^ TCID_50_/100 μl); each chick of control groups was injected with 100 μl DMEM. Three chickens were euthanized and the spleens, thymus, and bursa of fabricius were harvested on days 7, 15, 30, and 45 post infection of each group. Based on the immune index (the weight of spleen divided by the body weight), the spleens of chickens euthanized on days 15 were used for the DIA study. The DIA samples from were named as follows: the Pengxian chickens, Tibetan chickens, and Avian broiler treated with ALV-J were 15PI, 15TI, and 15AI, respectively. The Pengxian chickens, Tibetan chickens, and Avian broiler control groups were 15PC, 15TI, and 15AC, respectively. Statistical analyses were conducted via one-way ANOVA using SAS 8.0 software for Windows and the figures were made using GraphPad Prism 5.0. All data are expressed as mean ± SEM. P-values <0.05 were considered statistically significant.

### DIA procedures

The spleen protein digestion procedure was performed according to the literature, with minor modifications. The mixtures were placed into a Tissue Lyser for 2 min at 50 Hz to release proteins. After centrifugation with 25,000 g at 4°C for 20 min, the supernatant was transferred into a new tube, reduced with 10 mM dithiothreitol (DTT) at 56°C for 1 hand alkylated by 55 mM iodoacetamide (IAM) in the dark at room temperature for 45 min. Following centrifugation (25,000 g, 4°C, 20 min), the supernatant containing proteins was quantified by Bradford and sodium dodecyl sulfate-polyacrylamide gel electrophoresis (SDS-PAGE). Trypsin Gold (Promega, Madison, WI, USA) was used to digest the proteins at 37°C. The peptides were desalinated and vacuum dried. The peptides were separated on a Shimadzu LC-20AB HPLC Pump system coupled with a high pH RP column. The eluted peptides were pooled as 10 fractions and vacuum-dried. The peptides separated in liquid phase were ionized by nanoESI source and then entered the Q-Exactive HF (Thermo Fisher Scientific, San Jose, CA, USA) for DIA mode detection. This process was mainly based on a high-resolution mass spectrometer to produce sample data. For large-scale DIA data, the MaxQuant [16] and Spectronaut [17] used constructed spectrum image database information to complete the deconvolution extraction of data, and the mProphet algorithm was used to complete the analysis and quality control of data by Msstats software package [18]. The biological repetition of each group was executed the DIA procedure, respectively. Based on the quantitative results, differential proteins between different comparison groups were searched and functional analysis of differentially enriched proteins was performed by R software packages.

### Multiple reaction monitoring (MRM) procedures

Protein extraction, quality control, and enzymatic hydrolysis were carried out according to the above methods. Samples were digested as described and spiked with 50 fmol of β-galactosidase for data normalization. MRM analyses were performed on a QTRAP5500 mass spectrometer (AB SCIEX, Foster City, CA, USA) equipped with an LC-20AD nanoHPLC system (Shimadzu, Kyoto, Japan). A spectral library of MS/MS data was generated on a TripleTOF5600 (AB SCIEX, Foster City, CA, USA) and searched using Mascot v2.3 (Matrix Science, London, UK) against aHomo database (35985 entries). The data file was imported into Skyline software where a library was built. The peptides were selected for MRM method development according to the following criteria: (1) peptides with unique sequences in the database; (2) a maximum peptide m/z of <1250 (limitation of Quadrupole scan), with a peptide length range of 5–40 aa; (3) no Methionine in peptides; (4) Carbamidomethyl present on Cysteine and no variable modifications in peptides; and (5) no missed cleavage of trypsin. The chromatograms of all transitions generated on QTRAQP5500 were input to Skyline. The MRM method of a given protein was successfully developed only if the protein had at least one unique peptide which (1) was identified with the MS/MS spectral library (cutoff score >0.95), (2) had >5 fragment ions with the same elution profile and in the same ratios as the spectral library, and (3) had an accurate retention time (less than ± 2 min deviation from the predicted retention time). Statistical analysis was performed using one-way analysis of variance (ANOVA). P-values <0.05 were considered statistically significant.

## Results

### Disease statistics

**Figure 1.**
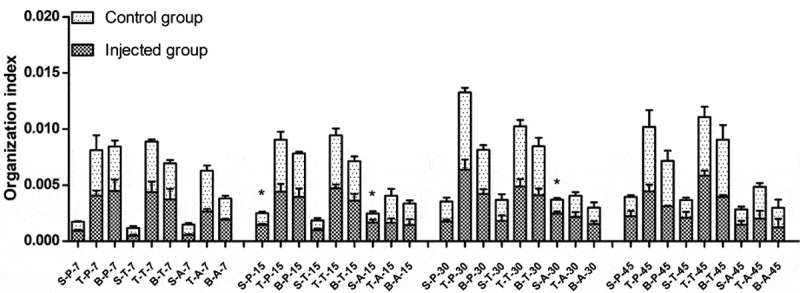
Organization index of infected and control groups of different breeds at different times. S: spleen; T: thymus; B: bursa of fabricius. P: Pengxian yellow chicken; T: Tibetan chicken; A: Avian broilers. All values are represented as the mean ± SEM (n = 3). (*) represents statistical significance (P < 0.05).

Organization indexes at different times in the injected and control groups of different breeds are shown in Figure 1. Since the NX0101 strains came from broiler breeds [15], susceptible breeds Avian broilers were added. On day 15, the organotin index of spleen function was significantly higher in both Pengxian yellow chickens and Avian broilers injected with ALV-J than in the control group of each breed (P < 0.05). On day 30, the organotin index of spleen function was significantly higher in Avian broilers injected with ALV-J than in the control group (P < 0.05). There were no significant differences in the thymus or bursa of fabricius among the three breeds on days 7, 15, 30, or 45 of injected with ALV-J groups and control groups. There were no significant differences in the spleen, thymus, or bursa of fabricius on days 7, 15, 30, or 45 in Tibetan chickens injected with ALV-J compared to Tibetan chickens in the control group.

### Identification of proteins

**Figure 2.**
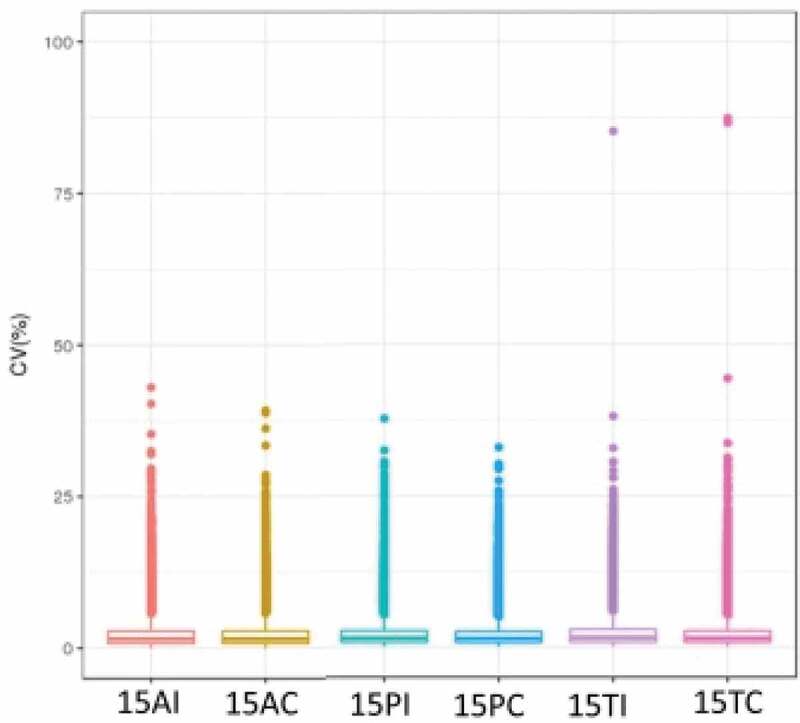
CV profile of each groups. The horizontal axis is the sample groups, and the vertical axis is the corresponding CV.

Approximately 30,000 peptides and 6,000 proteins were identified in each sample. Figure 2 shows the coefficient of variation (CV) within groups that was used to evaluate the quality of the data. The data had high reliability and could be further analyzed. Table 1 briefly summarizes the peptide number and protein number for each sample. Fold change ≥2 and P < 0.05 were used as the screening criteria for significantly differentially expressed proteins (DEPs). The results are shown in Table 2. There were 57 down-regulated proteins and 43 up-regulated proteins between the 15AC group and 15PC group, 233 down-regulated proteins, and 119 up-regulated proteins between the 15AC group and 15TC group, and 140 down-regulated proteins and 113 up-regulated proteins between 15PC group and 15TC group.

**Table 1.** Overview of quantitative results of each sample.

Sample name	Peptide number	Protein number
15AI-1	30210	5974
15AI-2	29323	5849
15AI −3	29737	5931
15AC-1	29197	5866
15AC-2	30960	6109
15AC-3	31248	6097
15PI-1	31876	6177
15PI-2	30839	6034
15PI −3	30817	6079
15PC-1	31172	6139
15PC-2	31329	6090
15PC-3	30889	6095
15TI-1	31494	6113
15TI-2	32305	6139
15TI −3	31516	6149
15TC-1	31979	6103
15TC-2	31274	6077
15TC-3	30931	6026

**Table 2.** Statistical list of differential proteins.

Comparison group	Down-regulated	Up-regulated	Non-regulated
15AC-vs-15PC	57	43	6257
15AC-vs-15TC	233	119	5915
15PC-vs-15TC	140	113	6051
15AI-vs-15AC	65	38	6159
15PI-vs-15PC	25	51	6301
15TI-vs-15TC	48	44	6255
15A-vs-15P	192	69	6277
15A-vs-15T	387	153	5914
15P-vs-15T	141	99	6257

A total of 65 down-regulated proteins and 38 up-regulated proteins between the 15AI group and 15AC group, 25 down-regulated proteins, and 51 up-regulated proteins between the 15PI group and 15PC group, and 48 down-regulated proteins and 44 up-regulated proteins between the 15TI group and 15TC group. There were 192 down-regulated proteins and 69 up-regulated proteins between the Avian broilers and Pengxian yellow chickens, 387 down-regulated proteins, and 153 up-regulated proteins between the Avian broilers and Tibetan chickens, and 141 down-regulated proteins and 99 up-regulated proteins between the Pengxian yellow chickens and Tibetan chickens.

### The analysis of DEPs in spleen development of three breeds

**Figure 3.**
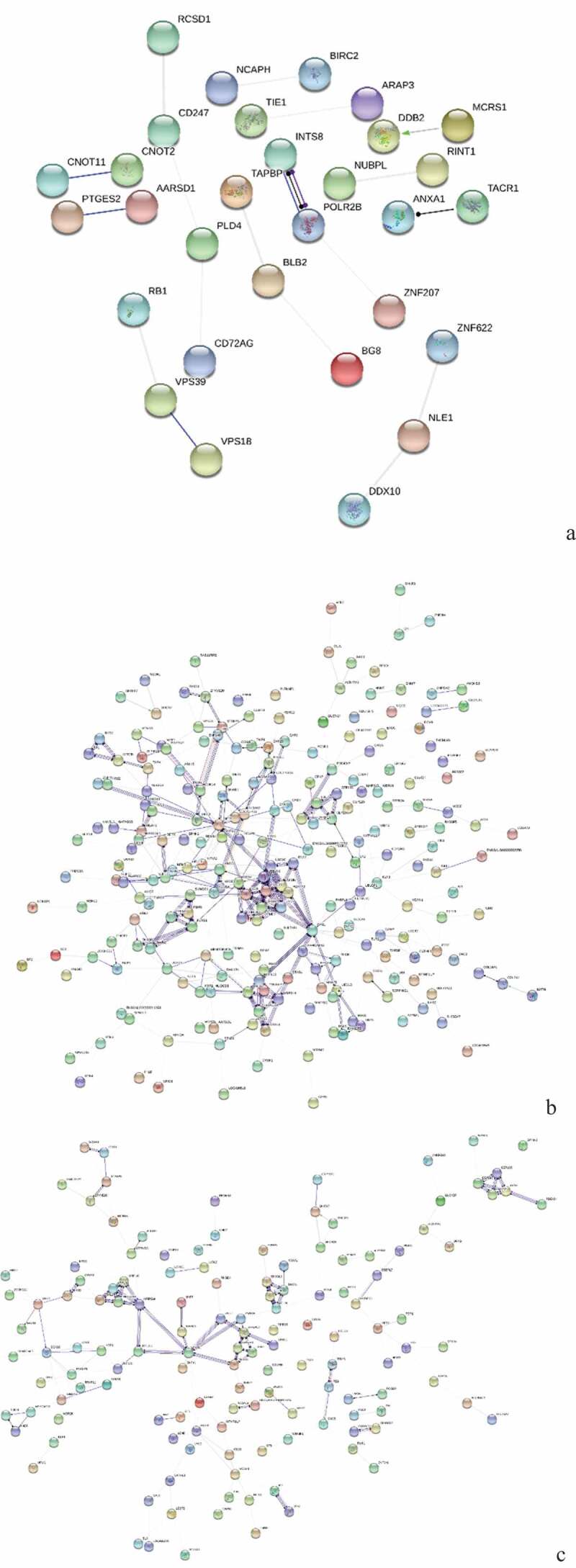
Protein interaction network analysis of DEPs of spleen development of three breeds. A: 15AC-VS-15PC; B: 15AC-VS-15 TC; C: 15PC-VS-15 TC. “ **–** ”activation; “ **–** ”inhibition; “ **–** ”binding; “ **–** ”catalysis; “ **–** ”phenotype; “ **–** ”posttranslational modification; “ **–** ”reaction; “ **–** ”transcriptional regulation; “→”positive; “**—**”negative; “ – **∙**”unspecified.

**Figure 4.**
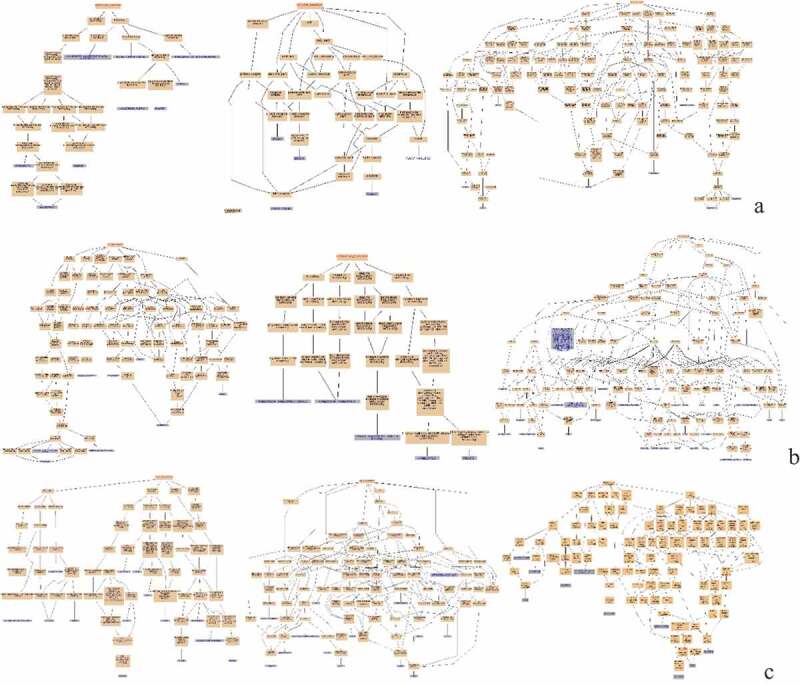
GO Enrichment analysis of DEPs in the spleens of three breeds. A: 15AC-VS-15PC; B: 15AC-VS-15 TC; C: 15PC-VS-15 TC. Cluster frequency means the ratio of annotation is the same GO term between all of DEPs and all of the proteins.

**Figure 5.**
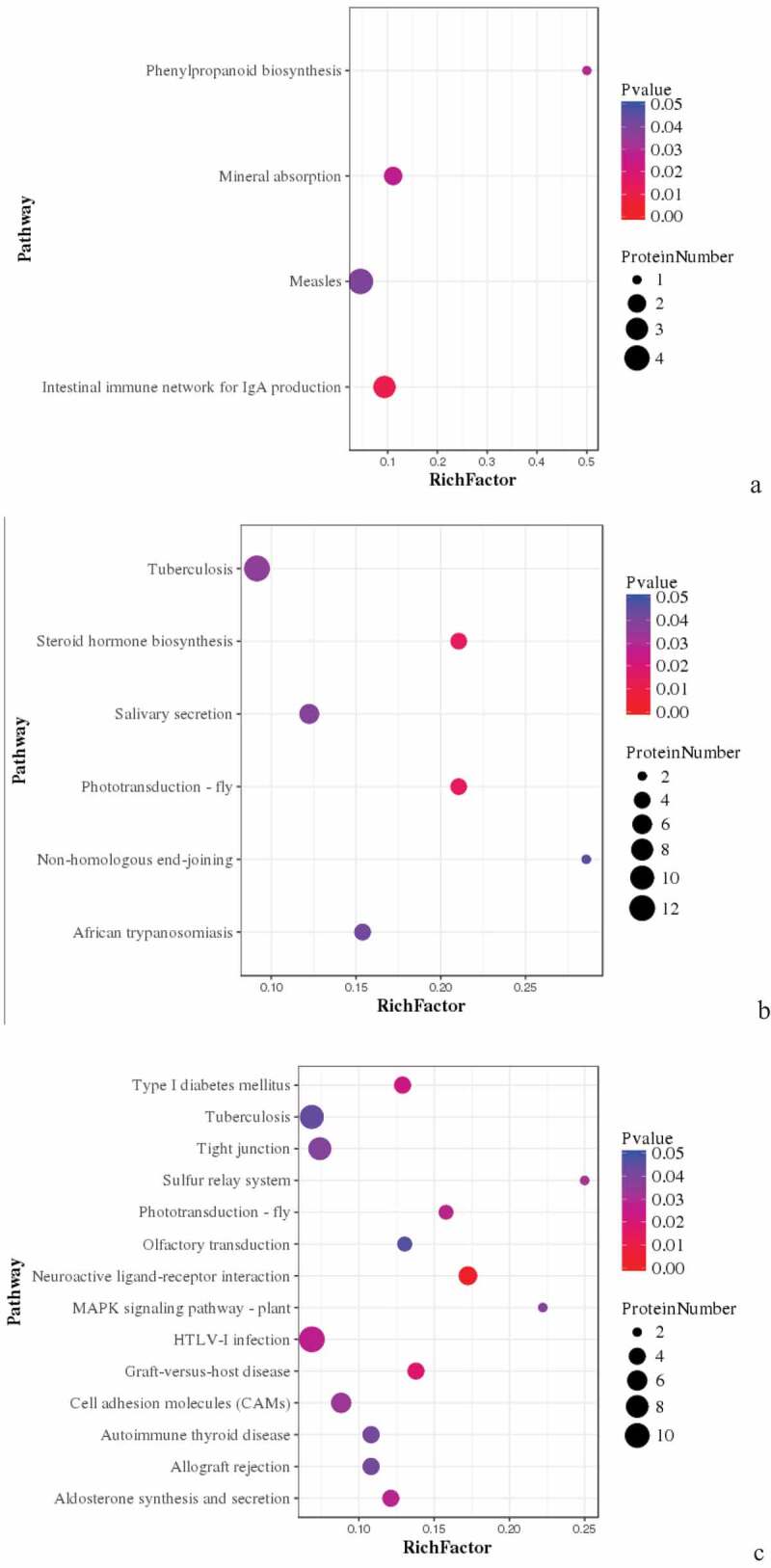
Pathway enrichment analyses of DEPs in spleens of three breeds. A: 15AC-VS-15PC; B: 15AC-VS-15 TC; C: 15PC-VS-15 TC. The enrichment factor is the number of DEPs annotated to the pathway divided by all of the identified proteins annotated to the pathway. The higher the value, the higher the proportion of differentially expressed proteins annotated to this pathway. The dot size in the figure represents the number of DEPs annotated to this pathway.

The three breeds were compared for spleen development analysis and the protein interaction network, GO function enrichment, and pathways of DEPs were analyzed. The results are shown in Figures 3, 4 and 5. Table 3 shows the DEPs of |log2FC| >3.

**Table 3.** Statistical list of pathway enrichment of differentially expressed proteins (DEPs) in the spleens of three breeds (|log2FC|>3).

Protein	Label	log2FC	Class	Description
E1C8D6	15AC-vs-15PC	3.323645	Up	HMG box transcription factor BBX-like
F1P450	15AC-vs-15PC	4.247857	Up	ras-related protein Rab-44
A0A097QQS6	15AC-vs-15TC	−3.41556	Down	pericentrin
E1C8R7	15AC-vs-15TC	−4.70327	Down	5ʹ-3ʹ exoribonuclease 1 isoform 2
Q5ZJL7	15AC-vs-15TC	−3.87885	Down	DNA damage-binding protein 2
Q31620	15AC-vs-15TC	−3.65289	Down	B-G, partial
A0A1D5PHB6	15AC-vs-15TC	−3.5766	Down	probable ATP-dependent RNA helicase DHX36 isoformX2
F1NXB9	15AC-vs-15TC	−3.49384	Down	conserved oligomeric Golgi complex subunit 3 isoform X2
E1C847	15AC-vs-15TC	−3.43345	Down	protein C16orf88
A0A1D5P4Q4	15AC-vs-15TC	−3.3724	Down	transcription factor p65
A0A1D5PJV2	15AC-vs-15TC	−3.14667	Down	epoxide hydrolase 3-like, partial
Q04584	15AC-vs-15TC	−3.06853	Down	Zyxin OS = Gallus gallus GN = ZYX
E1C8D6	15PC-vs-15TC	−4.23538	Down	HMG box transcription factor BBX-like
B5BSS3	15PC-vs-15TC	−3.69056	Down	MHC class I alpha chain 2
A0A1D5PHB6	15PC-vs-15TC	−3.64064	Down	probable ATP-dependent RNA helicase DHX36 isoformX2
A0A1D5PSQ6	15PC-vs-15TC	−3.51151	Down	cytochrome P450 2C19-like
A0A1D5P908	15PC-vs-15TC	−3.39676	Down	protein canopy homolog 3 isoform X1
E1C8R7	15PC-vs-15TC	−3.2081	Down	5ʹ-3ʹ exoribonuclease 1 isoform 2
A0A1D5P368	15PC-vs-15TC	−3.15845	Down	tRNA-dihydrouridine(16/17) synthase [NAD(P)(+)]-like
A0A097QQS6	15PC-vs-15TC	−3.00982	Down	pericentrin
F1NXW7	15PC-vs-15TC	4.787266	Up	WW domain-containing oxidoreductase
H9KYW7	15PC-vs-15TC	3.903517	Up	hyaluronidase-1
F1P0X4	15PC-vs-15TC	3.492125	Up	SWI/SNF related, matrix associated, actin dependent
A0A1D5P662	15PC-vs-15TC	3.30494	Up	chromodomain-helicase-DNA-binding protein 8-like, partial
F1NFQ4	15PC-vs-15TC	3.14415	Up	HAUS augmin-like complex subunit 2
A0A1D5NY42	15PC-vs-15TC	3.040815	Up	centrosomal protein of 135 kDa isoform X7

There were interactions between TACR1 and ANXA1, INTS8 and POLR2B, CNOT11 and CNOT2, and PTGES2 and AARSD1 of Avian broilers and Pengxian yellow chickens and DDB2 was positively regulated by MCRS1 (Figure 3 (A)). The spleen developmental DEPs of Avian broilers and Pengxian yellow chickens were formed interactions network by UBE2V2 and FBXL20 as the center, PCNT, HAUS2, and CEP290 as the center, MRPS7, ERAL1, and MRPS18A as the center, RBM5, PHF5A, and PLRG1 protein interactions as the center and CDK14, ZNF148, and ORC2 as the center. There was negative regulation or positive regulation between DEPs, such as LMO7 negatively regulated FBXL20 and KDM5A positively regulated NUP153 (Figure 3 (B)). The spleen developmental DEPs of Pengxian yellow chickens and Tibetan chickens were formed interactions network by MRPS14, OASL, and RNF7 as the center, CTTN, TACR1, and CLTA as the center and CEP290, PCNT and HAUS2 as the center. There was negative regulation or positive regulation between DEPs, such as TFDP1 negatively regulated RB1 and RB1 positively regulated SMC5 (Figure 3 (C)).

Figure 4 (A) shows the GO enrichments of DEPs in the spleen development of Avian broilers and Pengxian yellow chickens. DEPs were enriched in cell receptor complex, alpha-beta T cell receptor complex, HOPS complex (cellular component); histone acetyltransferase activity, phosphatidylinositol binding and carbohydrate binding (molecular function); regulation of signal transduction, fatty acid transport, and Notch signaling pathway (biochemical process).

Figure 4 (B) shows the GO enrichments of DEPs in the spleen development of Avian broilers and Tibetan chickens. DEPs were enriched in transcriptional repressor complex and nuclear origin of replication recognition complex (cellular component); serine-type endopeptidase inhibitor activity and low-density lipoprotein receptor activity (molecular function); convergent extension involved in axis elongation, histone H2A acetylation, and visual perception (biochemical process).

Figure 4 (C) shows the GO enrichments of DEPs in the spleen development of Pengxian yellow chickens and Tibetan chickens. DEPs were enriched in AP-1 adaptor complex, trans-Golgi network transport vesicle, and HAUS complex (cellular component); histone tyrosine kinase activity, protein tyrosine kinase activity, and stem cell factor receptor activity (molecular function); response to antibiotics, response to lipopolysaccharides, defense response to bacterium and response to inorganic substances (biochemical process).

KEGG pathway enrichment was analyzed for DEPs. DEPs involved in the intestinal immune network for IgA production, mineral absorption, and phenylpropanoid biosynthesis, such as BLB, Mx1, and RAB44 were compared in spleen development between Avian broilers and Pengxian yellow chickens (Table 3) (Figure 5 (A)). DEPs involved in tuberculosis, African trypanosomiasis and non-homologous end-joining, such as RAB44, VH1, MATN3, DDB2, and VCAM1 were compared in spleen development between Avian broilers and Tibetan chickens (Table 3) (Figure 5 (B)). DEPs involved in tuberculosis, neuroactive ligand–receptor interaction, MAPK signaling pathway, HTLV-I infection, graft-versus-host disease, autoimmune thyroid disease and allograft rejection, such as BF2, DHX36, CNPY3, and HYAL1 were compared in spleen development between Pengxian yellow chickens and Tibetan chickens (Table 3) (Figure 5 (C)).

### The analysis of DEPs of infected with ALV-J groups and control groups of three breeds

**Figure 6.**
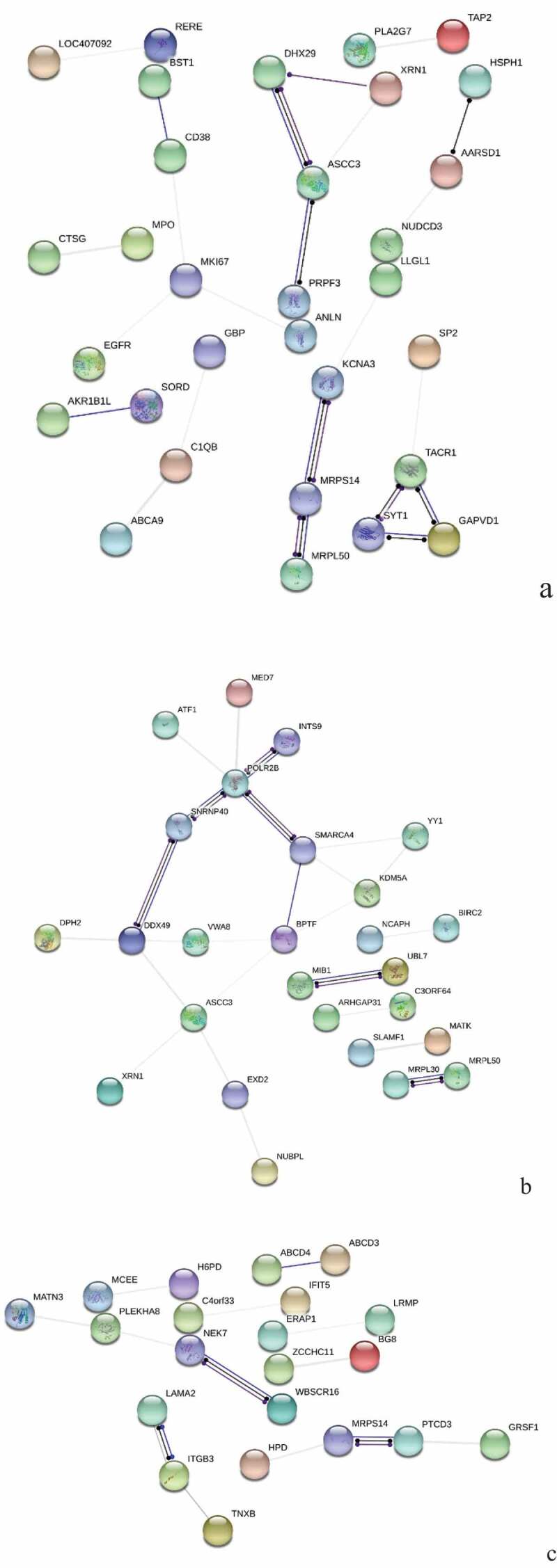
Protein interaction network analysis of DEPs of spleen of injected groups and control groups of three breeds. A: 15AI-VS-15AC; B: 15PI-VS-15PC; C: 15 TI-VS-15 TC. “ **–** ”activation; “ **–** ”inhibition; “ **–** ”binding; “ **–** ”catalysis; “ **–** ”phenotype; “ **–** ”posttranslational modification; “ **–** ”reaction; “ **–** ”transcriptional regulation; “→”positive; “**—**”negative; “ – **∙**”unspecified.

**Figure 7.**
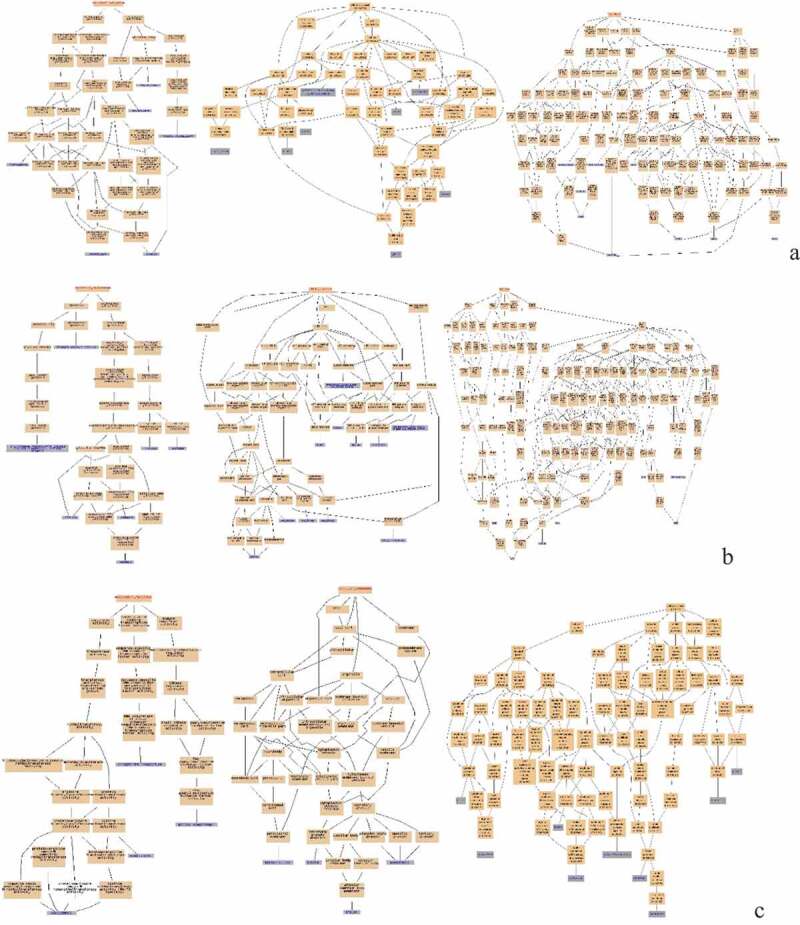
GO Enrichment analysis of DEPs in spleens of injected and control groups of three breeds. A: 15AI-VS-15AC; B: 15PI-VS-15PC; C: 15 TI-VS-15 TC. Cluster frequency means the ratio of annotation is the same GO term between all DEPs and all proteins.

**Figure 8.**
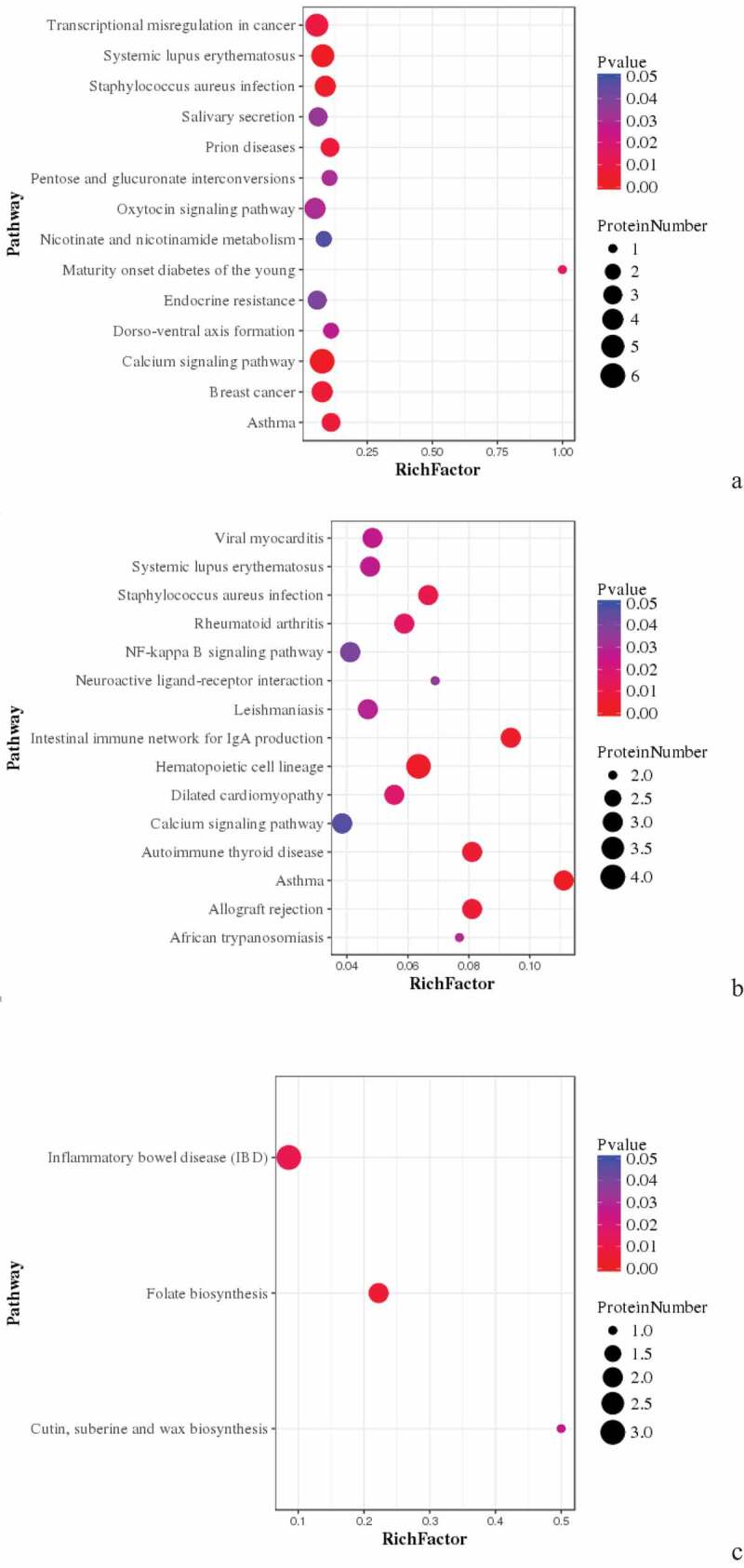
Pathway Enrichment Analysis of DEPs of spleens of injected and control groups of three breeds. A: 15AI-VS-15AC; B: 15PI-VS-15PC; C: 15 TI-VS-15 TC. The enrichment factor is the number of DEPs annotated to the pathway divided by all of the identified proteins annotated to the pathway. The higher the value, the higher the proportion of differentially expressed proteins annotated to this pathway. The dot size in the figure represents the number of DEPs annotated to this pathway.

The proteomics of infected with ALV-J groups and control groups of three breeds were compared. Protein interaction network, GO function enrichment, and pathway of DEPs were analyzed. The results are shown in Figures 6, 7 and 8. Table 4 shows the DEPs of |log2FC| >3.

**Table 4.** Statistical list of pathway enrichment of DEPs in spleens of three breeds after infection with ALV-J (|log2FC|>3).

Protein	Label	log2FC	Class	Description
R4GLC1	15AI-vs-15AC	3.24585	Up	CDC42 small effector protein 2
Q5F4B9	15TI-vs-15TC	3.62041	Up	ATP-binding cassette sub-family D member 3
A0A1D5P662	15TI-vs-15TC	3.63285	Up	chromodomain-helicase-DNA-binding protein 8-like, partial
A0A1D5PRS0	15TI-vs-15TC	3.7887	Up	WD repeat-containing protein 53
A0A1D5PCE8	15AI-vs-15AC	3.89955	Up	ensconsin
Q5ZJ90	15AI-vs-15AC	3.91162	Up	immunoglobulin-like receptor CHIR-AB1-like precursor
E1C8D6	15PI-vs-15PC	3.95761	Up	HMG box transcription factor BBX-like
F1NMZ3	15TI-vs-15TC	4.09283	Up	hemoglobin subunit epsilon
R4QXY1	15TI-vs-15TC	4.22519	Up	gag and reverse transcriptase polyprotein precursor
E1C479	15TI-vs-15TC	4.37746	Up	pleckstrin homology domain-containing family A member 8 isoform X2
F1P450	15PI-vs-15PC	4.42345	Up	ras-related protein Rab-44
E1C8R7	15AI-vs-15AC	4.45945	Up	5ʹ-3ʹ exoribonuclease 1 isoform 2
R4GLV4	15AI-vs-15AC	−4.179	Down	antigen KI-67 isoform X1
F1P450	15AI-vs-15AC	−4.0864	Down	ras-related protein Rab-44
F1NN75	15PI-vs-15PC	−3.9336	Down	lysine-specific demethylase 5A isoform X2

For the DEPs of Avian broilers infected with ALV-J groups and control groups, TACR1, SYT1, and GAPVD1 were interacted with each other, KCNA3, MRPS14, and MRPL50 were interacted with each other, and PRPF3, ASCC3, and DHX29 were interacted with each other (Figure 6 (A)). For the DEPs of Pengxian yellow chickens infected with ALV-J groups and control groups, there were interactions between DDX49, SNRNP40, POLR2B, and INTS9 proteins, UBL7 and MIB1 proteins, and MRPL50 and MRPL30 proteins (Figure 6 (B)). There were interactions between NEK7 and WBSCR16, MRPS14 and PTCD3, and LAMA2 and ITGB3 of the DEPs of Tibetan chicken infected with ALV-J groups and control groups (Figure 6 (C)).

Figure 7 (A) shows the GO enrichments of DEPs in infected with ALV-J groups and control groups of Avian broilers. DEPs were enriched in integral to plasma membrane protein, MHC class II protein complex and transport vesicle (cellular component); guanylate cyclase activity, ligand-gated sodium channel activity, and voltage-gated potassium channel activity (molecular function); regulation of protein transport, negative regulation of neurotransmitter secretion, and negative regulation of protein import into nucleus (biochemical process).

Figure 7 (B) shows the GO enrichments of DEPs in infected with ALV-J groups and control groups of Pengxian yellow chickens. DEPs were enriched in transcription factor complex, MHC class II protein complex, and integral to plasma membrane (cellular component); chromatin binding, ATP-dependent helicase activity, and DNA-dependent ATPase activity (molecular function); positive regulation of phagocytosis, negative regulation of growth of symbiont in host and positive regulation of viral transcription (biochemical process).

Figure 7 (C) shows GO the enrichments of DEPs in infected with ALV-J groups and control groups of Tibetan chickens. DEPs were enriched in secretory granule membrane, specific granule, and alveolar lamellar body membrane (cellular component); histone methyltransferase activity and Rho guanyl-nucleotide exchange factor activity (molecular function); regulation of exocytosis, regulation of gene expression by genetic imprinting and histone methylation (biochemical process).

KEGG pathway enrichment was analyzed for DEPs. DEPs in infected with ALV-J groups and control groups of Avian broilers were involved in transcriptional misregulation in cancer, salivary secretion, prion diseases, endocrine resistance, breast cancer, and asthma such as CDC42SE2, XRN1, MKI67, and RAB44 (Table 4) (Figure 8 (A)). DEPs in infected with ALV-J groups and control groups of Pengxian yellow chickens were involved in viral myocarditis, staphylococcus aureus infection, NFκB signaling pathway, intestinal immune network for IgA production, hematopoietic cell lineage, and autoimmune thyroid disease, such as KDM5A and RAB44 (Table 4) (Figure 8 (B)). DEPs in infected with ALV-J groups and control groups of Tibetan chicken were involved in inflammatory bowel disease and folate biosynthesis, such as PLEKHA8, HBBR, and ABCD3 (Table 4) (Figure 8 (C)).

### The analysis of DEPs of each pairwise comparison of three breeds after ALV-J infection

**Figure 9.**
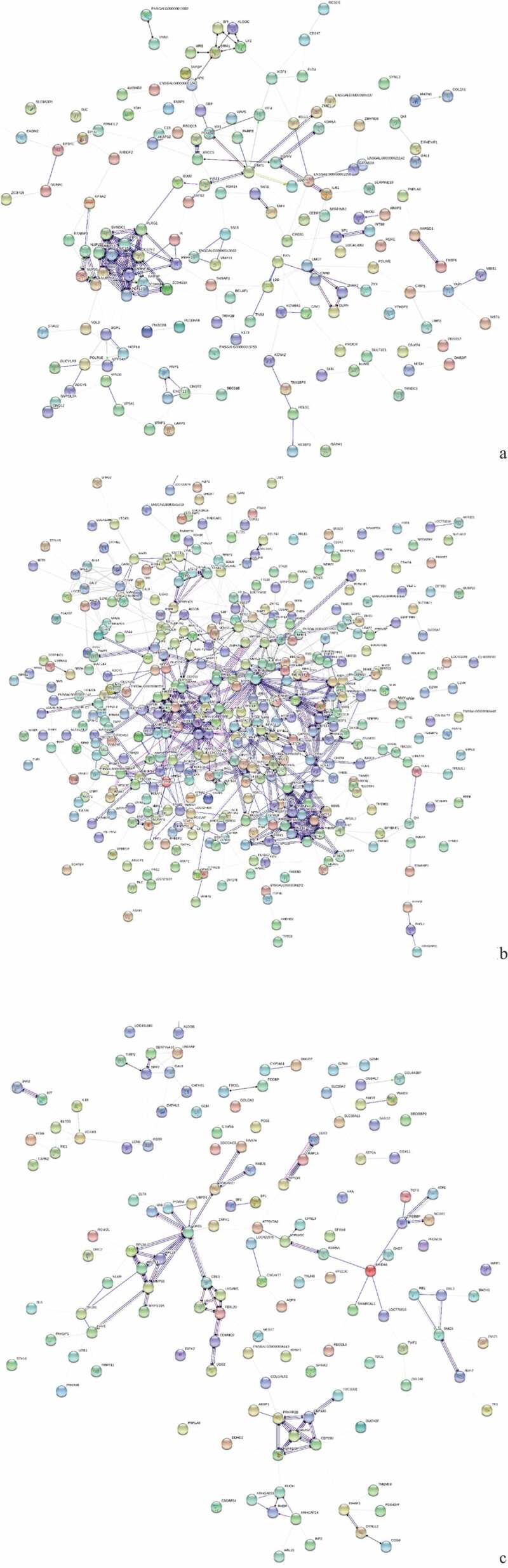
Protein interaction network analysis of DEPs of each comparison group. A: 15A-VS-15P; B: 15A-VS-15 T; C: 15P-VS-15 T. “ **–** ”activation; “ **–** ”inhibition; “ **–** ”binding; “ **–** ”catalysis; “ **–** ”phenotype; “ **–** ”posttranslational modification; “ **–** ”reaction; “ **–** ”transcriptional regulation; “→”positive; “**—**”negative; “ – **∙**”unspecified.

**Figure 10.**
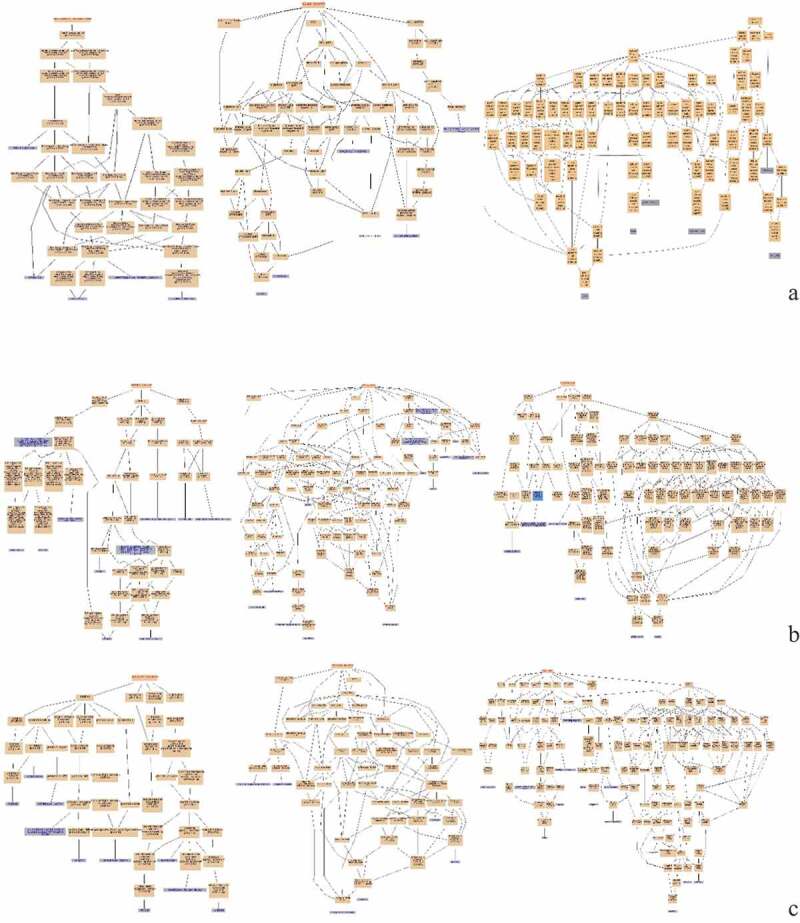
GO enrichment analysis of DEPs of each comparison group. A: 15A-VS-15P; B: 15A-VS-15 T; C: 15P-VS-15 T. Cluster frequency means the ratio of Annotation is the same GO term between all DEPs and all proteins.

**Figure 11.**
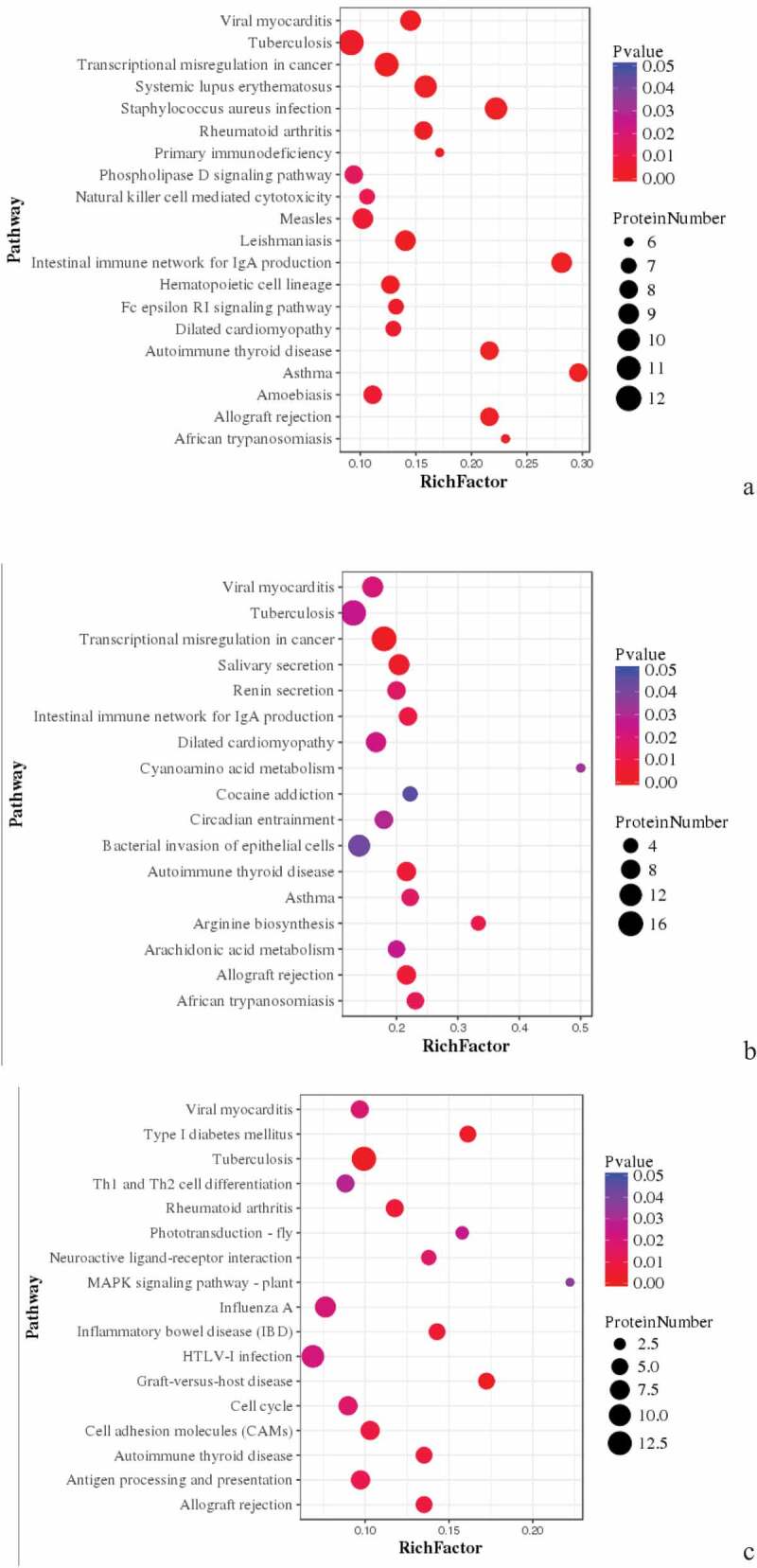
Pathway enrichment analyses of DEPs of each comparison group. A: 15A-VS-15P; B: 15A-VS-15 T; C: 15P-VS-15 T. The enrichment factor is the number of DEPs annotated to the pathway divided by all of the identified proteins annotated to the pathway. The higher the value, the higher the proportion of differentially expressed proteins annotated to this pathway. The dot size in the figure represented the number of DEPs annotated to this pathway.

Pairwise analysis was performed for the three breeds under the stimulation of ALV-J infection. Protein interaction network, GO functional enrichment, and pathway enrichment analysis were carried out for the obtained DEPs. The results are shown in Figures 9, 10 and 11. Table 5 shows the DEPs of |log2FC |> 3.

**Table 5.** Statistical list of pathway enrichment of DEPs in spleens of ALV-J infected comparable groups (|log2FC|>3).

Protein	Label	log2FC	Class	Description
H9L0X6	15A-vs-15T	3.059501	Up	59 kDa 2ʹ-5ʹ-oligoadenylate synthase-like protein
F1N917	15A-vs-15T	3.069164	Up	granzyme A precursor
A0A1D5NUT9	15A-vs-15T	3.071343	Up	calmodulin-regulated spectrin-associated protein 1 isoform X4
E1C224	15A-vs-15T	3.071702	Up	vacuolar protein sorting-associated protein 13 C isoform X1
F1NCJ9	15A-vs-15T	3.302237	Up	pseudouridylate synthase 7 homolog
A0A1D5P8V4	15A-vs-15T	3.461746	Up	retinoblastoma-binding protein 1
F1NMA2	15A-vs-15P	4.062002	Up	sulfotransferase 1 family member D1 isoform X2
F1NXW7	15A-vs-15T	4.110277	Up	WW domain-containing oxidoreductase
E1BQG2	15A-vs-15T	4.308023	Up	zinc finger protein 148 isoform X7
Q49LT3	15A-vs-15P	4.855398	Up	MHC class II B-L beta minor, partial
Q3L3M9	15P-vs-15T	−4.45211	Down	MHC class II antigen, partial
A0A1D5PFD1	15A-vs-15T	−4.39871	Down	ankyrin repeat domain-containing protein 27
A0A1D5PAP1	15P-vs-15 T	−3.88273	Down	echinoderm microtubule-associated protein-like 1 isoform X1
A0A1D5PRS0	15A-vs-15 T	−3.85457	Down	WD repeat-containing protein 53
Q6KDZ1	15A-vs-15 T	−3.82684	Down	basement membrane-specific heparan sulfate proteoglycan core protein precursor
A0A1D5PSQ6	15A-vs-15 T	−3.66235	Down	cytochrome P450 2C19-like
Q04584	15A-vs-15P	−3.5242	Down	zyxin
A0A1D5P4Q4	15A-vs-15 T	−3.45117	Down	transcription factor p65
P19753	15A-vs-15 T	−3.20125	Down	parvalbumin, thymic
E1C847	15A-vs-15 T	−3.19397	Down	protein C16orf88
F1NUK8	15A-vs-15P	−3.18916	Down	U5 small nuclear ribonucleoprotein 40 kDa protein

The CSTF2, FIP1L1, SMNDC1, PCF11, and other DEPs were the center of the interaction network of Avian broilers and Pengxian yellow chickens after ALV-J infection, and there was positive regulation between DEPs, such as NUP153, which had positive regulation on NUP35, and STAT1, which had positive regulation on IRF4 and CD274 (Figure 9 (A)). The interaction network of DEPs of Avian broilers and Tibetan chickens after ALV-J infection was complex, mainly centered on RAD51, ZNRF2, CBLB, OASL, CWC25, RBM5, etc., and there was positive or negative regulation among DEPs, such as DDB2 with positive regulation of STMN1 and EPB41L2 with negative regulation of RNF7 (Figure 9 (B)). The DEPs of Pengxian yellow chicken and Tibetan chicken after ALV-J infection were formed, respectively, by OASL, RPL19, RPL7L1 as the center of the interaction network, CBLB, UBR2, FBXL20 as the center of the interaction network, ARID4A, KDM5A, CREBBP as the center of the interaction network, and there was positive or negative regulation among DEPs, such as RBL2 positively regulated SMC5, CBLB negatively regulated OASL (Figure 9 (C)).

Figure 10 (A) shows the GO enrichments of DEPs of Avian broiler and Pengxian yellow chickens after ALV-J infection. DEPs were enriched in voltage-gated potassium channel complex, HOPS complex, and protein-DNA complex (cellular component); voltage-gated potassium channel activity, delayed rectifier potassium channel activity, and ligand-gated sodium channel activity (molecular function); negative regulation of endothelial cell differentiation, positive regulation by host of viral transcription and negative regulation of vascular endothelial growth factor signaling pathway (biochemical process).

Figure 10 (B) shows the GO enrichments of DEPs of Avian broilers and Tibetan chickens after ALV-J infection. DEPs were enriched in low-density lipoprotein particle, proteinaceous extracellular matrix, and extracellular vesicular exosome (cellular component); DNA replication origin binding, adenylate cyclase activity, and guanylate cyclase activity (molecular function); response to lipopolysaccharide, defense response to bacterium, defense response to fungus, positive regulation by host of viral transcription and negative regulation by host of viral transcription (biochemical process).

Figure 10 (C) shows the GO enrichments of DEPs of Pengxian yellow chickens and Tibetan chickens after ALV-J infection. DEPs were enriched in MHC class I protein complex, MHC class II protein complex, and AP-1adaptor complex (cellular component); DNA replication origin binding, stem cell factor receptor activity, and ATPase binding (molecular function); immunoglobulin mediated immune response, immune system process, innate immune response, interleukin-12-mediated signaling pathway and defense response to bacterium (biochemical process).

KEGG pathway enrichment was conducted for DEPs in each comparison group. DEPs in Avian broilers and Pengxian yellow chickens after ALV-J infection were involved in viral myocarditis, transcriptional misregulation in cancer, primary immunodeficiency, natural killer cell-mediated cytotoxicity, and intestinal immune network for IgA production such as ZYX, SNRNP40, and BLB (Table 5) (Figure 11 (A)). DEPs in Avian broilers and Tibetan chickens after ALV-J infection were involved in viral myocarditis, transcriptional misregulation in cancer, intestinal immune network for IgA production, B arterials invasion of epithelial cells and autoimmune thyroid disease such as ANKRD27, HSPG, RELA, and ZNF148 (Table 5) (Figure 11 (B)). DEPs in Pengxian yellow chickens and Tibetan chickens after ALV-J infection were involved in viral myocarditis, Th1 and Th2 cell differentiation, MAPK signaling pathway, inflammatory bowel disease, HTLV-I infection, graft-versus-host disease, autoimmune thyroid disease, and antigen processing and presentation such as BLBII, BF2, CREBBP, and NCOR1 (Table 5) (Figure 11 (C)).

### The DEPs detected by MRM

MRM technology was used to scan the partial DEPs of pairwise analysis results. Beta-galactosidase was used as the control protein and the data was naturalized with the intention of reducing the experimental error of MRM nonstandard quantification [19–21]. The experiment verified the difference in proteomics according to the scanning results, and the MRM results are shown in Table 6. As shown by the results, the MRM verification results were basically consistent with the results from the DIA results.

**Table 6.** Comparison of differential proteins from MRM and DIA.

Protein	Group	MRM results	DIA results	Conformity
Ig mu chain C region	B3-VS-P3	0.004661267	0.046437252	Yes
ALDOB	P3-VS-Z3	0.049635372	0.049419393	Yes
Parvalbumin, thymic	B3-VS-Z3	0.00156497	3.70E-05	Yes
LPP	B3-VS-P3	0.036017921	0.014830943	Yes
RBM14	B3-VS-P3	0.026782053	0.000466591	Yes
RBM14	B3-VS-Z3	0.11894563	0.000272064	No
PRSSL1	B3-VS-P3	0.019156264	0.0101381	Yes
PRSSL1	B3-VS-Z3	0.006123618	6.94E-05	Yes
Nucleoporin 214	B3-VS-Z3	0.029213517	0.01771383	Yes
SERPING1	B3-VS-Z3	9.08E-06	0.036030668	Yes
F1NA58	B3-VS-Z3	0.012647483	0.018285934	Yes
RAP1A	P3-VS-Z3	0.053604991	0.008348641	No
BPI	B3-VS-P3	0.024581122	0.001148476	Yes
BPI	B3-VS-Z3	0.001779427	0.004130038	Yes
HPX	B3-VS-P3	0.014573999	0.004703354	Yes
HPX	B3-VS-Z3	0.002213361	0.011606742	Yes

## Discussion

Genetic selection is considered a feasible and reliable method for improving immunity in chickens. China’s local chicken genetic resources are rich and provide a lot of material for breeding disease resistance. In order to select chicken breeds with higher disease resistance, it is necessary to first understand the relevant molecular mechanisms, so as to explore the selection of markers related to disease resistance.

The Pengxian yellow chicken has a round and medium-sized body; the Tibetan chicken has a light, small, long, low, symmetrical, and compact body; and the Avian broiler has full, heavy, wide, deep body. ALV-J is an RNA retrovirus, which has a complex pathogenic mechanism and high variability. The effective methods to improve the resistance of breeds to the virus is the genetic breeding. According to the disease statistics, the Tibetan chicken has the strongest resistance to ALV-J, followed by the Pengxian yellow chicken and then the Avian broiler, which is the most sensitive, which showed that local chickens have great potential as candidates for disease resistance breeding.

Protein function is a dynamic biological process. Proteomics is the macroscopic detection of changes in protein expression through omics technology, which is conducive to the in-depth study of biological processes. Proteomics analysis is a powerful and relatively new technique for studying biomarkers of protein response to viral infection and has been used in chickens [22]. Chickens with different genetic backgrounds have different levels of proteins during development. In the current study, the phenotype of the two local breeds (Pengxian yellow chicken and Tibetan chicken) were confirmed to be different from the Avian broiler after ALV-J infection, which might be related to the different development of immune organs in the three breeds. DIA technology was used to analyze proteomics in the spleens of 15-day-old healthy hens from three different breeds and the specific proteins of each breed were detected, such as PCNT, DBI, CATHL1, MHCI, VCAM1, and IGF2BP1 of Tibetan chicken, PCNT, RBM14, HNMT, TFEB, CD247, and DDB2 of Avian broilers, ZNF622, TAF10, TPN, SNX13, TIE1, ASPR, and HINT2 of Pengxian yellow chickens. These DEPs were involved in tissue components (lysosomes, cell receptor complexes, and transcription factor complexes), metabolism (fatty acid transport, arachidonic acid secretion, and nucleotide biosynthesis), signal transmission (acetylation and groups of protein tyrosine kinase activity), and immune response (Notch signal pathway, lipopolysaccharide reaction, and defense). The biological functions enriched with different proteins led to the differences in spleen development among the three breeds which specific effect on virus resistance still needs further study of virus infection.

AL molecular markers are difficult to study due to the complex mechanisms involved in ALV-J infection and the complicated immune mechanisms in chickens. Only a few studies have used proteomics to study molecular markers in AL [12–14]. Chickens are susceptible to ALV-J and the spleen plays an important role in anti-infection and immune response function in viral infection. In this study, the index of spleens of Tibetan chickens infected with ALV-J was no significantly different from control groups, while the index of spleens of Pengxian yellow chicken and Avian broilers infected with ALV-J was significantly higher than control groups of each breed. Proteomics analysis of spleens was conducted in each breed infected with ALV-J groups and control groups. Using proteomics techniques, there were no common DEPs in three breeds of infected with ALV-J groups and control groups, but MKI67, RAB44, ELP4, HINT2, ASCC3, MRPL50, and XRN1 were both detected in Pengxian yellow chickens and Avian broilers infected with ALV-J groups and control groups, and BLB, TPN, PRMT7, NSUN5, DDX20, TRAF3, NFATC1, and NFIX were detected in Tibetan chicken infected with ALV-J groups and control groups. These DEPs were enriched in inflammatory bowel disease, antigen processing and presentation, Th17 cell differentiation, toll-like receptor signaling pathway, TNF signaling pathway, and other signaling pathways related to the immune response. The differences in proteins among the three breeds showed that the infection mechanism of ALV-J is complex and is associated with different genetic backgrounds. These DEPs may be potential markers of spleen immune response after ALV-J infection in chickens.

The application of proteomics technology in ALV-J infection provides a new way to explore the mechanism of ALV-J infection and the immune capacity of the body at a macro level. After taking into account normal genetic background differences among the three breeds, DEPs were analyzed between three breeds after ALV-J infection. The proteins RFX1, VCAM1, PRMT7, TCF, GNMT, CATHL1, MHCI, TRMT11, and CD48 of Tibetan chickens were differently expressed with Pengxian yellow chickens and Avian broilers. The proteins TAF10, KDM5A, SFN, OSBPL7, CNOT11, and PNPLA6 of Pengxian yellow chicken were differently expressed with Tibetan chicken and Avian broilers. The proteins VH1, IRF4, MHCII, LBP, CD247, PIAS1, ERG, CREB, YTHDF3, YTHDF2, BCR, and STAT1 of Avian broilers were differently expressed with Tibetan chicken and Pengxian yellow chickens. These DEPs are involved in cancer transcriptional dysregulation, IgA production of intestinal immune network, TNF signaling pathway, IL-17 signaling pathway, toll-like receptor signaling pathway.

Vascular cell adhesion molecule 1 (VCAM1, also known as CD106) belongs to the immunoglobulin (Ig) superfamily of cell surface proteins [23,24]. VCAM1 is highly expressed in acute myeloid leukemia (AML) cells [25]. AML is one of the diseases of avian leukemia caused by ALV-J [2]. The up-regulated expression of VCAM1 in Tibetan chickens infected with ALV-J revealed that VCAM1 plays an important role in resistance to ALV-J infection in chickens; thus, VCAM1 could be a potential immune marker. Transcription factor X1 (RFX1) is a widely expressed dual active transcription factor that can activate and inhibit target genes. RFX1 is important in regulating the epigenetic state of T cells [26]. Deficiency of RFX1 promotes CD4 + T cells to differentiate into Th17 cells, which can be reversed by forcing the expression of RFX1 [27]. ALV-J induced immunosuppression is related to T cell differentiation [2]. The high expression of RFX1 in Tibetan chicken indicates that RFX1 may be related to the immunosuppression caused by ALV-J. RFX1 can play an anti-cancer role by down-regulating the original oncogene c-MYC [28]. The pathogenic mechanism caused by the insertion of ALV-J nucleic acid into host DNA is related to c-MYC [29]. Avian leukemia caused by ALV-J is a neoplastic disease. According to the function of RFX1 in cancer tumors, it could be that RFX1 may have an anti-virus function in ALV-J infection. The high expression of RFX1 in Tibetan chickens infected with ALV-J indicates that RFX1 plays a role in inhibiting the virus in ALV-J infection, which is consistent with the results found in other studies of RFX1. RFX1 could be used as a candidate marker molecule for poultry resistance to ALV-J infection. T cytokine 3 (TCF3, also known as E2A) is closely related to human acute lymphoid leukemia [30]. TCF3 protein is highly expressed in Tibetan chicken, suggesting that TCF3 may play the same role as RFX1 in suppressing the immune function of the virus. A host defense peptide (CATHL1) is an important component of innate immunity and can activate innate and adaptive immunity [31]. ALV-J induces immune response of the body [32–34]. The high expression of CATHL1 in Tibetan chicken indicates that CATHL1 plays an important role in anti-virus. Previous studies have shown the great potential of CATHL1 as an antiviral infectant [35], which is consistent with the results of this study. Major histocompatibility class I molecules (MHCI) are essential for host–pathogen interactions and deliver both their own and external antigenic peptides to T lymphocytes [36]. Studies on MHCI in poultry mainly focus on the relationship between its haplotype and resistance [37–39]. In this study, the expression of MHCI protein in Tibetan chickens was lower than in Avian broilers and Pengxian yellow chickens, indicating that MHCI protein may not play a major role in anti-virus. B cell antigen receptor (BCR) regulates B cell development by mediating the selection of functional and self-tolerant of B cells, thus ensuring immune protection and avoiding autoimmunity [40,41]. In chronic lymphocytic leukemia, BCR signal is in an abnormal state of activation [42]. In the current study, Avian broilers were sensitive to ALV-J and BCR was up-regulated, indicating that BCR could be used as a potential molecular marker of ALV-J infection. MHCII is a class II major histocompatibility complex involved in antigen presentation, restricted recognition between immune cells, T cell differentiation, and genetic control of the immune response [43–45]. The expression of MHCII protein was up-regulated in Avian broilers, down-regulated in Pengxian yellow chickens, and no significant difference in Tibetan chickens. This may be related to the different genetic background of Avian broilers, Pengxian yellow chickens, and Tibetan chickens. MHCII protein can be used as a potential marker molecule of ALV-J infection, but it is not suitable to be used as an evaluation factor of interbreed immunity. Lipopolysaccharide binding protein (LBP) is an acute protein synthesized in the liver. LBP and LPS complexes bind to CD14 expressed by macrophages and neutrophils and mediate signal transduction, including activation of NFκB via TLR4, which activates innate and adaptive inflammatory responses [46,47]. The interaction between LBP and CD14/TLR4 is directly involved in the activation of LPS-mediated function and the expression of innate immune genes in chicken [48]. The expression of LBP protein in Avian broilers was up-regulated; therefore, LBP protein could be used as one of the potential immune marker factors after ALV-J infection. Transcription regulator cAMP response element-binding protein (CREB) is a factor involved in the regulation of various cellular processes. IL-10 is the most important anti-inflammatory cytokine and CREB stimulates the transcription of IL-10 [49]. The expression of CREB protein in Avian broilers was down-regulated compared with the two other breeds, indicating that CREB protein could be used as a potential immune marker molecule after ALV-J infection.

## Conclusion

In this study, DIA technology was used to detect the DEPs of three breeds of chicken according to different comparison to investigate the potential markers. Special DEPs for spleen development of each breed were detected, such as PCNT, DDB2, and ZNF62. These DEPs were involved in intestinal immune network used in the production of IgA signaling pathways and related to immune response which can be used as potential markers for spleen development in different breeds. The DEPs such as RAB44 and TPN involved in viral myocarditis, transcriptional misregulation in cancer, and tuberculosis can be used as potential markers of spleen immune response after ALV-J infection in chickens. Pair-wise analysis was performed for the three breeds after the infection of ALV-J. The proteins such as RFX1, TAF10 and VH1 were differently expressed between three breeds. These DEPs involved in antigen processing and expression, acute myelogenous leukemia, and viral carcinogenesis can be used as potential immune markers after ALV-J infection of different genetic backgrounds.
